# Empirical study of seven data mining algorithms on different characteristics of datasets for biomedical classification applications

**DOI:** 10.1186/s12938-017-0416-x

**Published:** 2017-11-02

**Authors:** Yiyan Zhang, Yi Xin, Qin Li, Jianshe Ma, Shuai Li, Xiaodan Lv, Weiqi Lv

**Affiliations:** Department of Biomedical Engineering, School of Life Science, Beijing Institute of Technology, 5 South Zhongguancun Street, Haidian District, Beijing, 100081 China

**Keywords:** Classification task, Characters of datasets, Applicability of algorithm, Data mining

## Abstract

**Background:**

Various kinds of data mining algorithms are continuously raised with the development of related disciplines. The applicable scopes and their performances of these algorithms are different. Hence, finding a suitable algorithm for a dataset is becoming an important emphasis for biomedical researchers to solve practical problems promptly.

**Methods:**

In this paper, seven kinds of sophisticated active algorithms, namely, C4.5, support vector machine, AdaBoost, k-nearest neighbor, naïve Bayes, random forest, and logistic regression, were selected as the research objects. The seven algorithms were applied to the 12 top-click UCI public datasets with the task of classification, and their performances were compared through induction and analysis. The sample size, number of attributes, number of missing values, and the sample size of each class, correlation coefficients between variables, class entropy of task variable, and the ratio of the sample size of the largest class to the least class were calculated to character the 12 research datasets.

**Results:**

The two ensemble algorithms reach high accuracy of classification on most datasets. Moreover, random forest performs better than AdaBoost on the unbalanced dataset of the multi-class task. Simple algorithms, such as the naïve Bayes and logistic regression model are suitable for a small dataset with high correlation between the task and other non-task attribute variables. K-nearest neighbor and C4.5 decision tree algorithms perform well on binary- and multi-class task datasets. Support vector machine is more adept on the balanced small dataset of the binary-class task.

**Conclusions:**

No algorithm can maintain the best performance in all datasets. The applicability of the seven data mining algorithms on the datasets with different characteristics was summarized to provide a reference for biomedical researchers or beginners in different fields.

## Background

Massive data collection, storage, and fast delivery are simplified with the development of science and the innovation of technology. A large amount of data contains considerable valuable information, especially in the biomedical field. Data mining is an essential tool in understanding the value of a dataset. A wide variety of data mining methods has emerged with the prosperity of big data. However, their application scopes and focuses are slightly inconsistent. Thus, researchers are required to find a relatively optimal data mining method to promptly solve practical problems. Consequently, we aim to provide several suggestions to biomedical researchers by comparing seven active algorithms applied to the 12 top-click UCI public datasets with the task of classification.

Similar works in the field of data mining were unpopular. In the late 1990s, two European spirit projects, namely, StatLog [1] and METAL [2], were conducted successively. The StatLog project mainly concluded that no single best algorithm exists, and symbolic algorithms were favorable choices in maximizing accuracy when the distribution of data is extreme. METAL aims to develop model selection and method combination approaches that focus on classification and regression problems to provide users with an online environment support. The comparison of different candidate algorithms in the context of a specific application was recommended because the performances of machine learning algorithms were proven to be problem-dependent [3]. Certain research was conducted in the field of time series [4] or bioinformatics [5], which have the distinct characteristics of time variation or high dimension. Elmahgiubi [6] developed a generic meta-learning framework for automatic algorithm selection and then applied and evaluated the generic framework to solve the selection problem of packet classification algorithm. Limited studies for general dataset without significant macroscopic characteristics were conducted after the two European spirit projects to assess the applicability of algorithms. Lim et al. [7] claimed that the quick unbiased efficient statistical tree and logistic regression algorithms were substantially fast. According to the research of Ali and Smith [8], the classifier C4.5, the neural network, and the support vector machine (SVM) were all competitive as the best choices in terms of measurement accuracy. In addition, certain researchers focused on the ensemble of several base classifiers [9] or the overall workflow in certain software [10, 11], which provided the final result. These kinds of ensembles appeared as a type of black box model for users. Luo [12] reviewed the literature on automatic selection methods for machine learning algorithms and hyper-parameter values for a given supervised machine learning problem. He found that these methods have limitations in the extensive environment of biomedical data.

**Fig. 1:**
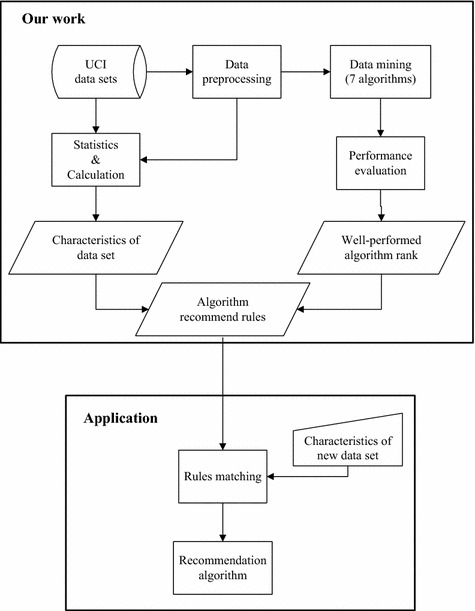
A general view of the work and application scenarios

In this study, seven active mature algorithms were selected to analyze their applicability to large real-world problems. To the best method for assessing the empirical applicability of these algorithms on large real-world problems is using large real-world data. The UCI machine learning repository [13] is a collection of databases, domain theories, and data generators that are used by machine learning communities for the empirical analysis of machine learning algorithms. The UCI machine learning repository is used by students, educators, and researchers worldwide as a primary source of machine learning datasets. Therefore, 12 top-click datasets, namely, “Iris,” “Adult,” “Wine,” “Car evaluation,” “Breast cancer Wisconsin,” “Wdbc,” “Wpbc,” “Abalone,” “Wine quality_red,” “Wine quality_white,” “Heart disease,” and “Poker hand,” with the task of classification from the UCI machine learning repository were selected as our research datasets. As previously described, the inclusion criteria of seven algorithms are state-of-the-art, high maturity and representative. The inclusion criteria of 12 datasets are real-world data, classic (or high usage), diversity. The applicable data mining methods of the 12 datasets with different characteristics were obtained through induction and analysis. The present study aims to provide a reference for biomedical workers with different backgrounds on method selection and scheme design. The working methodology and application scenarios are illustrated in Fig. 1.

The rest of this paper is organized as follows. “Methods” section describes in detail the quantitative index and research algorithms. Furthermore, “Results” section illustrates the obtained quantitative results and displays the performance assessments of the algorithms. “Discussion” section presents the further analysis and discussion. Finally, “Conclusion” section provides the comprehensive conclusions.

## Methods

### Quantitative index of dataset characteristics

Study plans are used to quantify or describe the characteristics of a dataset using seven quantitative indices; these indices include sample size, number of variables, correlation coefficient between target and other variables, correlation coefficient between non-target variables, class entropy of the target variable, number of missing values, and the ratio between the sample size of the largest class and the sample number of the least class. These quantitative indices represent the size of the dataset, dimension of the dataset, degree of correlation between variables, dispersion degree of the target variable, integrity of information, and the balance of the dataset.

### Data mining algorithm selection and algorithm summary

The algorithms, namely, C4.5 [14], SVM [15], AdaBoost (AB) [16], k-nearest neighbor (kNN) [17], naïve Bayes (NB) [18], random forest (RF) [19], and logistic regression (LR) model [20], are selected as our base algorithms using the criteria of maturity, representativeness, and activeness at present. Their applicability is discussed subsequently by comparing and contrasting these algorithms in detail.

The C4.5 algorithm, as one of the three classical decision tree algorithms, is derived from iterative dichotomizers 3. This algorithm can provide a mapping between attribute values and classification, and the mapping can be used to classify new unknown instances through learning [14]. SVM is one of the most robust and accurate approaches among all well-known data mining algorithms. SVM is based on the statistical learning theory and mainly includes support vector classification (SVC) and support vector regression. Boosting is the most important “family” of ensemble methods, in which the AB algorithm is one of the most important. AB without certain foreknowledge continuously updates weights in multiple iterations to achieve the optimal result in the learning process [21]. The kNN algorithm is an instance-based and lazy learning method. In particular, kNN does not process a training data until the prediction stage [22]. The NB algorithm is one of the oldest formal classification methods. A rule that can determine the category of an unknown object and only contains known vector without known category [23] is formulated according to Bayes’ theorem. The RF algorithm is a kind of ensemble learning method that can be applied to classification, regression, and outlier detection. RF is composed of decision trees without pruning. The LR model, as a concrete form of the generalized linear model, originates from the statistical community, and its link function is the logit function [20].

### Performance assessment of algorithms

Classification accuracy and running speed are mainly considered in the study to compare the performances of the different algorithms applied to the same dataset. Consequently, the concept of sensitivity and specificity is extended, that is, one of the classes is considered the focus class for a multi-class target variable, and the rest of the classes are combined. Then, the sensitivity and specificity of the current focus class can be computed to inspect the specific prediction accuracy of the current algorithm. User time is selected as the running time of the algorithm on the model building phase. In addition, this study monitors the memory usage of each algorithm prediction model to provide a reference for considering and discussing the simplicity of these models.

All the analyses are implemented using R software (R Foundation for Statistical Computing, Vienna, Austria) version 3.2.2 on a personal computer equipped with Intel Core i5-2400 CPU @ 3.10 GHz processor and Windows 7 operating system. The following R packages have been used: ‘e1071’, ‘RWeka’, ‘adabag’, ‘kknn’ ‘randomForest’ and ‘nnet’. Both SVM and NB were implemented under the framework of ‘e1071’ package with R. C4.5, AB, kNN, RF and LR were implanted under the framework of packages ‘RWeka’, ‘adabag’, ‘kknn’, ‘randomForest’ and ‘nnet’, respectively. Tuning parameters will have a significant impact on the performance of the algorithm. To reduce the interference of tuning parameters, for various parameters within the learning algorithms, default values were assigned as in the R package settings.

## Results

### Overview of datasets

Table 1 displays the basic statistical information of the 12 research datasets. Eleven datasets belong to the life, social, physical, business, and game areas, which were clearly marked on the shared link page of the dataset. The “Car evaluation” dataset has no division on the page. Thus, its area is excluded in Table 1.

**Table 1:** Profile of research data sets

Name of dataset	Sample size	Number of attributes	Missing values?	Task	Area
Iris	150	4	No	Multi-class	Life
Adult^a^	32,561	13	Yes	Binary-class	Social
Wine	178	13	No	Multi-class	Physical
Car evaluation	1728	6	No	Multi-class	–
Breast cancer Wisconsin^a^	699	9	Yes	Binary-class	Life
Wdbc^a^	569	30	No	Binary-class	Life
Wpbc^a^	198	31	Yes	Binary-class	Life
Abalone	4177	8	No	Multi-class	Life
Wine quality_red^a^	1599	11	No	Multi-class	Business
Wine quality_white^a^	4898	11	No	Multi-class	Business
Heart disease^a^	303	13	Yes	Multi-class	Life
Poker hand^a^	25,010	10	No	Multi-class	Game

^a^The dataset ‘Adult’ is a subset of the database ‘Adult Data Set’. The datasets ‘Breast cancer Wisconsin’, ‘Wdbc’ and ‘Wpbc’ are three subsets come from the same database ‘Breast Cancer Wisconsin (diagnostic) data set’. The datasets ‘Wine quality_red’ and ‘Wine quality_white’ are included in the same database ‘Wine Quality Data Set’. Limited to data quality, ‘processed.cleveland’ and ‘poker-hand-training-true’ two subsets were selected as represents of the databases ‘Heart Disease Data Set’ and ‘Poker hand data set’, respectively

As can be seen from Table 1, there are six datasets belong to ‘Life’ area, the biomedical data that we concern about.

### Quantification of dataset characteristics

The sample size, number of attributes, number of missing values, and the sample size of each class in the 12 research datasets are counted. The correlation coefficient between task and other non-task attribute variables, correlation coefficient between each couple non-task attribute variables, class entropy of task variable, and the ratio of the sample size of the largest class to the least class are calculated. The “Wine quality_red” dataset is considered an example. The quantitative results in the dataset characteristics are displayed and described.

Table 2 presents the correlation coefficients among the variables in the “Wine quality_red” dataset. Only the values of the low triangular matrix are listed because the correlation coefficient matrix is a symmetric matrix. By comparison, the absolute value of the correlation coefficient between the task variable and the variable “alcohol” is 0.4762, which is the maximum. The absolute value of the correlation coefficient between variables “fixed.acidity” and “pH” is the largest, and the value of the correlation coefficient is − 0.6830. The two values (0.4762 and − 0.6830) are included in the quantification index of dataset characteristics.

**Table 2:** Correlation coefficients between variables in ‘wine quality_red’ data set

	Fixed.acidity	Volatile.acidity	Citric.acid	Residual.sugar	Chlorides	Free.sulfur.dioxide	Total.sulfur.dioxide	Density	pH	Sulphates	Alcohol	Quality
Fixed.acidity	1	–	–	–	–	–	–	–	–	–	–	–
Volatile.acidity	− 0.2561	1	–	–	–	–	–	–	–	–	–	–
Citric.acid	0.6717	− 0.5525	1	–	–	–	–	–	–	–	–	–
Residual.sugar	0.1148	0.0019	0.1436	1	–	–	–	–	–	–	–	–
Chlorides	0.0937	0.0613	0.2038	0.0556	1	–	–	–	–	–	–	–
Free.sulfur.dioxide	− 0.1538	− 0.0105	− 0.061	0.187	0.0056	1	–	–	–	–	–	–
Total.sulfur.dioxide	− 0.1132	0.0765	0.0355	0.203	0.0474	0.6677	1	–	–	–	–	–
Density	0.668	0.022	0.3649	0.3553	0.2006	− 0.0219	0.0713	1	–	–	–	–
pH	− 0.683	0.2349	− 0.5419	− 0.0857	− 0.265	0.0704	− 0.0665	− 0.3417	1	–	–	–
Sulphates	0.183	− 0.261	0.9128	0.0055	0.3713	− 0.0517	0.0429	0.1485	− 0.1966	1	–	–
Alcohol	− 0.0617	− 0.2023	0.1099	0.0421	− 0.2211	− 0.0694	− 0.2057	− 0.4962	0.2056	0.0936	1	–
Quality	0.1241	− 0.3906	0.2264	0.0137	− 0.1289	− 0.0507	− 0.1851	− 0.1749	− 0.0577	0.2514	0.4762	1

In Table 3, no missing value is observed in the “Wine quality_red” dataset. The task variable contains six classes, indicating that the dataset is multi-class. The sample size of the largest and least class is 681 and 10, respectively. The dataset is an imbalanced dataset, which has huge differences among the sample sizes of the different classes.

**Table 3:** Quantification of the characteristics of ‘Wine quality_red’ dataset

Quantification index	Values
Sample size	1599
Number of attributes	11
Number of missing values	0
Number of classes	6
Sample size of the largest class	681
Sample size of the least class	10
Correlation coefficients1^a^	0.4762
Correlation coefficients2^a^	− 0.6830
Class entropy of task variable	0.5145
Ratio of sample size of the largest class to the least class	68.10

^a^Correlation coefficients1 represents the maximum of correlation coefficient between task variable and other non-task attribute variables; correlation coefficients2 represents the maximum of correlation coefficient between each pair of non-task attribute variables

### Evaluation and quantification of the performances of the algorithms

The C4.5, SVM, AB, kNN, NB, RF, and LR models are implemented on each research dataset to conduct classification prediction. Then, the prediction results are evaluated to compare the performances of the aforementioned algorithms. Table 4 displays the evaluation results when “Wine quality_red” is considered an example.

**Table 4:** Performance evaluation of the algorithms applied to ‘Wine quality_red’ dataset

Algorithm	Accuracy	Sensitivity (Class ‘3’)	Sensitivity (Class ‘5’)	Specificity (Class ‘3’)	Running time (s)	Memory usage (M)
C4.5	0.9099	0.8000	0.9266	0.9956	0.15	0.02
SVM	0.6717	0	0.8062	1.0000	0.79	0.53
AdaBoost	0.6629	0	0.7871	1.0000	34.02	11.33
kNN	0.8705	0.7000	0.9178	1.0000	0.11	0.39
Naïve Bayes	0.5604	0.3000	0.6696	0.9975	0.00	0.01
Random forest	1.0000	1.0000	1.0000	1.0000	1.42	10.33
Logistic regression	0.6079	0.2000	0.7518	0.9981	0.23	0.34

**Fig. 2:**
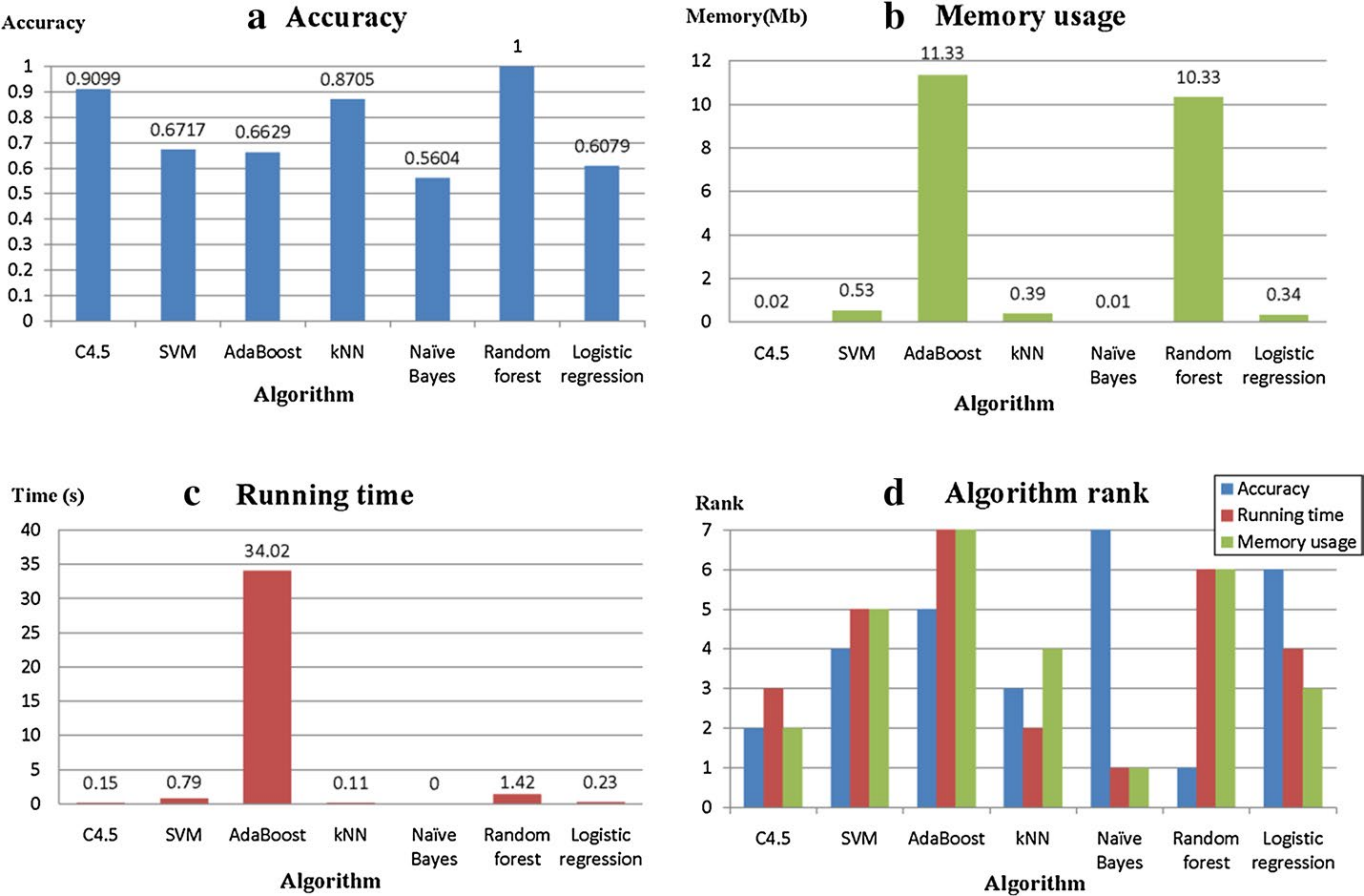
Evaluation and rank of algorithms on the ‘Wine quality_red’ data set

In Table 4, certain hints about the algorithms can be found based on the prediction accuracy aspect. The RF algorithm performs well on the “Wine quality_red” dataset, and the C4.5 algorithm demonstrates the best performance among the single classifiers. The NB nearly shows no cost, and AB is significantly overloaded. The NB exhibits a significant superiority and occupies a limited memory. Furthermore, the observed space usage of C4.5 is minimal. However, two ensemble algorithms, namely, RF and AB, occupy large memory. Figure 2 depicts the comparison results.

Figure 2 demonstrates the accuracy, running time, memory usage, and the rank of the seven algorithms. In Fig. 2a, the RF algorithm performs well and exhibits the best result among the algorithms. In Fig. 2b, the RF suggests a poor performance with a value of 10.33 Mb in terms of memory usage. Similar interpretations can be derived in the running time aspect. In Fig. 2c, the RF demonstrates the second longest running time. Figure 2d illustrates the rank of the seven algorithms in selecting an optimal algorithm quickly and intuitively. In Fig. 2d, no algorithm can be declared for all criteria on the “Wine quality_red” dataset. The RF is ranked first in accuracy but lags behind in running time and memory usage. On the contrary, the NB is ranked first in terms of running time and memory usage but is the last in terms of accuracy.

### Comparison and induction of multiple dataset results

The characteristic quantification values and performance assessment of the algorithms applied to the 12 research datasets for comparison and analysis are summarized in Table 5. The sample size in Table 1 is the initial number of samples, and the sample size in Table 5 is the number of samples after the deletion of the missing values. The target variable is not included in the number of attributes. Cor1 represents the maximum of the correlation coefficient between task and other non-task attribute variables. Moreover, Cor2 denotes the maximum of the correlation coefficient among all pairs of non-task attribute variables. AB and RF are collectively called “Ensemble.” The “Single classifier” corresponds to the other five algorithm models, excluding the two ensemble methods in the seven research algorithms.

**Table 5:** Characteristic quantification values and performance assessment of algorithms applied to the 12 research datasets

Dataset	Sample size	Number of attributes	Number of classes	Cor1	Cor2	Class entropy	Balance	Well-performed algorithm rank
Iris	150	4	3	0.9565	0.9629	0.4771	1	Ensemble, single classifier
Adult	30,162	13	2	0.3353	− 0.5849	0.2437	3.017	Ensemble, C4.5
Wine	178	13	3	− 0.8475	0.8646	0.4717	1.479	Ensemble, LR, SVM, other
Car evaluation	1728	6	4	0.4393	0	0.3630	18.62	Ensemble,C4.5, kNN^a^
Breast cancer Wisconsin	683	9	2	0.8227	0.9072	0.2812	1.858	Ensemble, kNN, C4.5, SVM
Wdbc	569	30	2	0.7936	0.9979	0.2868	1.684	Ensemble,LR, C4.5, kNN, SVM
Wpbc	194	31	2	− 0.3460	0.9959	0.2379	3.217	Ensemble, C4.5, kNN^a^
Abalone	4177	8	28	0.6276	0.9868	1.084	689	RF, kNN, C4.5
Wine quality_red	1599	11	6	0.4762	− 0.6830	0.5145	68.1	RF, C4.5, kNN
Wine quality_white	4898	11	7	0.4356	0.8390	0.5604	439.6	RF^b^, C4.5, kNN
Heart disease	297	13	5	0.5212	0.5790	0.5577	12.31	RF, kNN, AB, C4.5
Poker hand	25,010	10	10	0.0102	− 0.0303	0.4277	2498.6	kNN, C4.5

‘Other’ in the last column means remaining algorithms besides previous listed algorithms

^a^kNN has higher sensitivity on a certain class, namely kNN has higher accuracy when predict the certain class

^b^RF occupied bigger memory, then 2000 instances were sampled randomly to be training set, and RF showed high classification accuracy and acceptable running speed

## Discussion

We can obtain the following rules based on the results listed in Table 5.

- The two ensemble methods, namely, AB and RF, exhibit outstanding classification accuracy on most datasets. Moreover, RF performs better than AB. However, the ensemble methods spend an increased time and require an enhanced operating environment for a large dataset with thousands of samples because of hardware limitations.
- Nearly no difference is observed among the seven algorithms for a small dataset with high correlation between the task and other non-task attribute variables.
- On the binary-class balanced dataset, SVM performs better than that in the multi-class dataset. That is, SVM is suitable for the balanced dataset with the binary-class task.
- The kNN and C4.5 algorithms show remarkable performance in the binary- and multi-class datasets. Furthermore, kNN outperforms C4.5 algorithm in datasets with further classes and large sample size.
- NB behaves well in datasets with small sample size, a minimal number of classes, and high correlation between variables. The NB with dataset exhibiting other characteristics reveals inferior performance than the other six algorithms.
- SVM occupies more memory than the other single classifier algorithms and has higher requirements for operating environment.

According to the results of this study, the recommended algorithms of datasets with different characteristics are summarized in Table 6.

**Table 6:** Summary of applicative algorithm recommendation on different characteristic datasets

Character of dataset	NB	LR	kNN	C4.5	SVM	AB	RF	Represents of dataset
Small sample size	√	√				√	√	Iris, wine
High correlation	√	√						Iris, wine
Binary-class task		√			√	√		Breast cancer Wisconsin, Wdbc
Balanced data	√	√			√			Wine, breast cancer Wisconsin, Wdbc
Multi-class task			√	√			√	Abalone, wine quality_red
Imbalanced data			√	√			√	Wine quality_white
Large sample size			√				√	Adult, poker hand
Low correlation			√	√		√	√	Car evaluation, Wpbc, heart disease

### No free lunch (NFL) theorem

In the machine learning context, the NFL theorem [24] implies that all learning algorithms perform equally well when averaged among all possible datasets. No method can exceed random guessing without additional information assistance. Therefore, the forms of classifiers are completely determined by a priori information or numerous training samples. Moreover, many data types indicate diverse underlying data structures during various practical problems. The adjustment of the decision boundary to adapt to these structures is crucial for the classification problems, especially the generalization ability of the classifiers. Our experimental results prove the theorem that no algorithm can maintain the best performance in all datasets.

### NB algorithm

Occam’s razor principle highlights that the most simple model or hypothesis should be selected preferentially when two models have the same predictive effect and efficiency. The potential implication of this scenario is that this model is insignificant when a model structure is more complex than the original dataset [25]. Thus, simple algorithms, such as the NB and LR models, should be adopted for a small dataset with high correlation between the task and other non-task attribute variables if different algorithms demonstrate minimal differences in prediction accuracy, similar to the biology dataset “Iris.” This phenomenon not only ensures the accuracy of the model but also simplifies its complexity. The NB model is generally effective. Its prominent advantage is its simple computation, especially for a discrete variable model. Notably, this model is widely applied because it is easy to understand and explain [21]. A relatively new important application of NB is spam filtering.

Missing data is a potential problem in nearly all data analyses. Since some algorithms are sensitive to missing values, we delete the missing values in each data set when performing data preprocessing. The ratio of the missing values in the paper is a term used to describe the characteristic of the dataset. Particularly, the missing value is often happened in medical data. Most classification algorithm cannot control missing data. If data are missing at random, then NB does not encounter any difficulty in processing because the marginal distribution can be effectively estimated from the observed data. However, this process becomes complex in the missing data of information type, and thus this field requires further research [21].

In bioinformatics, the “large p, small n” problem (small sample size but high dimension) is crucial. This problem is common in genomics, proteomics, and microarray data analysis. The characteristic of the problem is that the number of variables is much larger than that of the sample, resulting in the ill-conditioned problem of singular covariance matrix and overfitting. The introduction of several assumptions or the reduction of the estimator equivalently in certain ways is necessary to overcome these problems. One of the available methods to manage the problem in a supervised classification task is to use the NB model. This built-in assumption can effectively resist overfitting because of independence. The obtained classifier becomes complex when the skillful improvement ideas are enhanced. Therefore, seeking the best balance between these aspects should be considered [21]. The research datasets of this paper do not contain bioinformatics-related data, such as genomics and proteomic data. The datasets with this kind of special structure will be included in our further study.

### LR model

The LR model is more powerful but requires more complex estimation framework than the NB model. The parameter estimation in the LR model cannot be simply estimated using proportion, and iterative algorithm must be used. The datasets “Wine” and “Wdbc” are characterized by few classes, high correlation, and rough balance. The LR model performs well on the two datasets. The dependent variable of the LR model can be binary or multiple, but the binary-class is commonly used and easily explained. For example, the LR model is commonly applied to explore the risk factors that cause disease and predict the probability of disease occurrence according to the risk factors. The LR model is widely used in medicine, banking, and marketing. The LR model yields low computational cost and is easy to understand and implement. By contrast, the LR model can be underfitting, and the classification accuracy may be low.

### kNN and C4.5

In this study, the kNN and C4.5 decision tree algorithms perform well on binary- and multi-class task datasets. In Table 5, kNN and C4.5 appear as one of the well-performed algorithms on nearly all datasets. The kNN algorithm performs better than the C4.5 algorithm for datasets, such as “Abalone” and “Poker hand,” with large number of classes and sample size. Cover and Hart contended that the classification error rate of the kNN rules under certain conditions will not be more than two times the optimal Bayesian error rate. Furthermore, the error rate of the kNN method is asymptotically convergent to the Bayesian error rate; thus, the kNN method can be used as an approximation of the Bayesian method under normal circumstances [26]. The kNN classifier saves modeling time compared with active learning methods, such as decision tree and SVM. However, kNN consumes additional time on classifying unknown objects because it requires calculating the kNN of the object. Thus, for some urgent situations, for example, the prediction of adverse cardiac events, atrial fibrillation, kNN does not apply. The kNN classification is easy to understand and implement and performs well in many cases, such as classifying article and other texts [27] and predicting spatial data-like satellite images [28]. Researchers on the study of gene function allocation based on microarray expression found that kNN is superior to SVM [29]. However, the C4.5 algorithm cannot only induce the decision tree but also convert the decision tree into rules with well intelligibility. The earliest decision trees only manage categorical data; recently, they are extended to support numerical, symbolic, and mixed data types. Similarly, the decision tree application fields, such as clinical decision making, manufacturing, document analysis, bioinformatics, and geographic information system, is extensive. In practice, the C4.5 algorithm should be considered, provided that the interclass boundary of the target problem can be determined by a tree-splitting or rule-discriminating pattern.

### SVM

SVM is based on statistical learning theory. SVM can only select minimal training data from considerable training data for model building. For the linear and divisible binary-class learning task, SVC divides two classes of samples by finding the hyperplane with maximum margin. The maximum margin can ensure the best generalization ability of the hyperplane [30]. One disadvantage of the early SVM is that the computational complexity of the training phase is high, which may lead to inapplicability of algorithms in large-scale datasets. However, this problem has been solved [21]. In Table 5, the medical dataset “Breast cancer Wisconsin” is a representative of the balanced small dataset of the binary-class task. In our study, the SVM performance on this dataset type remains better than that of the multi-class large dataset. The result further provides a theoretical support for the above research findings [29].

### AB and RF

If a single classifier is a weak learner, then this classifier becomes a strong learner to assemble numerous single classifiers. The ensemble algorithm is a strong classifier, which is composed of one or more types of base classifiers. The performance of several single classifiers is inadequate for certain datasets; ensemble algorithms can be adopted if time and hardware conditions permit. AB and its variants are widely used in various fields because of its solid theoretical foundation, accurate prediction, and simple algorithm; these algorithms are gaining considerable success. For example, a strong face detector is obtained by combining AB and the face detection algorithm through the cascade process [31]. In the present study, “Ensemble,” as a well-performed algorithm, appears seven times, while RF appears four times in Table 5. Therefore, the two ensemble algorithms reach high accuracy of classification on most datasets. Moreover, RF performs better than AB on the unbalanced dataset of the multi-class task, such as datasets “Wine quality_red,” “Wine quality_white,” and “Heart disease.” Bischl et al. [32] emphasized that random regression forests emerged quite clearly as the best overall approach, achieving the best performance on 13 of the 17 datasets. RF can handle prodigious dimensionality, although numerous variables exist. Each base classifier only contains part of the randomly selected variables; therefore, deleting the variables is unnecessary. The RF model not only can manage the non-linear and non-Gauss problem but also demonstrate high prediction accuracy. In addition, the RF model provides two methods of measuring the importance of variables. RF is widely used in medicine, marketing, physics, and archaeology. However, a serious defect of the ensemble learning methods is its lack of intelligibility. These methods become a black-box model after ensemble, even when the base learner is an understandable model (such as small decision trees). Thus, improving the intelligibility of the ensemble learning method is a crucial research direction. But for some early detection of tumors, this type of black box model is still applicable. For example, doctors only need early detection and removal of intestinal polyps, without knowing their synergies and taking other treatment plan.

### Generalization ability and intelligibility

In the generalization ability, the algorithm not only exhibits a favorable classification performance on the training dataset but also can accurately predict the new data with uniform distribution of the training data. The intelligibility of the algorithm model is crucial in many practical applications. The two aspects should be considered in future research by seeking appropriate quantitative index to describe and evaluate. Ali et al. [33] introduced the quality meta-metrics (QMM) of algorithms. QMM can be used to describe the physical meaning of the evaluation criteria. The researchers developed a classification model to assist experts in selecting the suitable evaluation criteria for comparing classifiers using extensive literature. In our future work, we will consider this idea to satisfy different requirements.

## Conclusions

In the wave of big data, people gradually focus on collecting and utilizing data in all walks of life. However, experts from various fields are confused in selecting or applying data mining algorithms given the limitation in academic background knowledge. In this study, the applicability of each algorithm is obtained through the comparative analysis of seven kinds of mature algorithms on the classification task for datasets. For the balanced small biomedical dataset of the binary-class task, SVM is recommended to perform predict. The kNN and C4.5 decision tree algorithms perform well on binary- and multi-class task biomedical datasets. Moreover, C4.5 is easy to understand and interpret. The applicability rules can provide a reference in selecting data mining algorithms to biomedical researchers without scientific and engineering backgrounds. Thus, we are required to develop a reasonable plan combined with relevant background knowledge and select the appropriate mining methods to analyze or explore the potential knowledge in a large biomedical data. Then, the results should be discussed from a professional viewpoint. Finally, the rules or conclusions consistent with the actual condition can be obtained. Our results provide the possibility for combining expert knowledge with data mining methods and analysis tools. The results of this study show that attempting a variety of algorithms or selecting the proper algorithm for data mining can be accomplished promptly, and biomedical researchers can exert further efforts in learning and mastering the professional knowledge in their fields.

## Abbreviations

SVM: support vector machine
AB: AdaBoost
kNN: k-nearest neighbor
NB: naïve Bayes
RF: random forest
LR: logistic regression
SVC: support vector classification
NFL: no free lunch
QMM: quality meta-metrics

## References

[1] King RD, Feng C, Sutherland A (1995). Statlog: comparison of classification algorithms on large real-world problems. Appl Artif Intell.

[2] Smith-Miles KA (2009). Cross-disciplinary perspectives on meta-learning for algorithm selection. ACM Comput Surv.

[3] Heremans S, Orshoven JV (2015). Machine learning methods for sub-pixel land-cover classification in the spatially heterogeneous region of flanders (belgium): a multi-criteria comparison. Int J Remote Sens.

[4] Adhikari R (2015). A mutual association based nonlinear ensemble mechanism for time series forecasting. Appl Intell.

[5] Ding Y, Tang S, Liao SG, Jia J, Oesterreich S, Lin Y (2014). Bias correction for selecting the minimal-error classifier from many machine learning models. Bioinformatics.

[6] Elmahgiubi M. An efficient framework for automatic algorithm selection using meta-learning [D]. Guelph: University of Guelph; 2016.

[7] Lim TS, Loh WY, Shih YS (2000). A comparison of prediction accuracy, complexity, and training time of thirty-three old and new classification algorithms. Mach Learn.

[8] Ali S, Smith KA (2006). On learning algorithm selection for classification. Appl Soft Comput J.

[9] Cruz RMO, Sabourin R, Cavalcanti GDC, Ren TI (2015). Meta-des: a dynamic ensemble selection framework using meta-learning. Pattern Recogn.

[10] Soares C, Souza BFD (2014). Metastream: a meta-learning based method for periodic algorithm selection in time-changing data. Neurocomputing.

[11] Nguyen P, Hilario M, Kalousis A (2014). Using meta-mining to support data mining workflow planning and optimization. J Artif Intell Res.

[12] Luo G (2016). A review of automatic selection methods for machine learning algorithms and hyper-parameter values. Netw Model Anal Health Inform Bioinform.

[13] Lichman M. UCI Machine Learning Repository [http://archive.ics.uci.edu/ml]. Irvine, CA: University of California, School of Information and Computer Science. 2013.

[14] Quinlan JR (1993). C4.5: programs for machine learning.

[15] Cortes C, Vapnik V (1995). Support-vector networks.

[16] Freund Y., Schapire Robert E (1997). A desicion-theoretic generalization of on-line learning and an application to boosting. J Comput Syst Sci.

[17] Altman NS (1992). An introduction to kernel and nearest-neighbor nonparametric regression. Am Stat.

[18] Russel S, Norvig P (2010). Artificial intelligence—a modern approach. Appl Mech Mater.

[19] Breiman L (2001). Random forests. Mach Learn.

[20] Walker SH, Duncan DB (1967). Estimation of the probability of an event as a function of several independent variables. Biometrika.

[21] Wu X, Kumar V (2009). The top ten algorithms in data mining.

[22] Aha DW, Kibler D, Albert MK (1991). Instance-based learning algorithms. Mach Learn.

[23] Berger JO. Statistical decision theory and Bayesian analysis, vol. 83, no. 401. New York: Springer; 2011. p. 266.

[24] Wolpert DH (1996). The lack of a priori distinctions between learning algorithms. Neural Comput.

[25] Blumer A, Ehrenfeucht A, Haussler D, Warmuth MK (1987). Occam’s razor. Inf Process Lett.

[26] Cover T, Hart P (1967). Nearest neighbor pattern classification. IEEE Trans Inf Theory.

[27] Hmeidi I, Hawashin B, El-Qawasmeh E (2008). Performance of knn and svm classifiers on full word arabic articles. Adv Eng Inform.

[28] Mcinerney DO, Nieuwenhuis M, Mcroberts RE, Donoghue DNM, Deshayes M (2009). A comparative analysis of k NN and decision tree methods for the Irish national forest inventory. Int J Remote Sens.

[29] Kuramochi M, Karypis G (2001). Gene classification using expression profiles: a feasibility study. Int J Artif Intell.

[30] Wu X, Kumar V, Quinlan JR, Ghosh J, Yang Q, Motoda H (2008). Top 10 algorithms in data mining. Knowl Inf Syst.

[31] Viola P, Jones MJ (2001). Robust real-time object detection. Int J Comput Vision.

[32] Bischl B, Kerschke P, Kotthoff L, Lindauer M, Malitsky Y, Fréchette A (2016). Aslib: a benchmark library for algorithm selection. Artif Intell.

[33] Ali R, Lee S, Chung TC (2017). Accurate multi-criteria decision making methodology for recommending machine learning algorithm. Expert Syst Appl.

